# Complement Factor H and Apolipoprotein E Participate in Regulation of Inflammation in THP-1 Macrophages

**DOI:** 10.3389/fimmu.2018.02701

**Published:** 2018-11-21

**Authors:** Eija Nissilä, Pipsa Hakala, Katarzyna Leskinen, Angela Roig, Shahan Syed, Kok P. M. Van Kessel, Jari Metso, Carla J. C. De Haas, Päivi Saavalainen, Seppo Meri, Angeliki Chroni, Jos A. G. Van Strijp, Katariina Öörni, Matti Jauhiainen, T. Sakari Jokiranta, Karita Haapasalo

**Affiliations:** ^1^Department of Bacteriology and Immunology, and Research Programs Unit, Immunobiology, University of Helsinki, Helsinki, Finland; ^2^Medical Microbiology, University Medical Center Utrecht, Utrecht, Netherlands; ^3^Minerva Foundation Institute for Medical Research, Helsinki, Finland; ^4^Institute of Biosciences and Applications, National Center for Scientific Research “Demokritos”, Athens, Greece; ^5^Wihuri Research Institute, Helsinki, Finland

**Keywords:** complement, complement system, Factor H, apolipoprotein E, atherosclerosis, inflammation

## Abstract

The alternative pathway (AP) of complement is constantly active in plasma and can easily be activated on self surfaces and trigger local inflammation. Host cells are protected from AP attack by Factor H (FH), the main AP regulator in plasma. Although complement is known to play a role in atherosclerosis, the mechanisms of its contribution are not fully understood. Since FH via its domains 5–7 binds apoliporotein E (apoE) and macrophages produce apoE we examined how FH could be involved in the antiatherogenic effects of apoE. We used blood peripheral monocytes and THP-1 monocyte/macrophage cells which were also loaded with acetylated low-density lipoprotein (LDL) to form foam cells. Binding of FH and apoE on these cells was analyzed by flow cytometry. High-density lipoprotein (HDL)-mediated cholesterol efflux of activated THP-1 cells was measured and transcriptomes of THP-1 cells using mRNA sequencing were determined. We found that binding of FH to human blood monocytes and cholesterol-loaded THP-1 macrophages increased apoE binding to these cells. Preincubation of fluorescent cholesterol labeled THP-1 macrophages in the presence of FH increased cholesterol efflux and cholesterol-loaded macrophages displayed reduced transcription of proinflammatory/proatherogenic factors and increased transcription of anti-inflammatory/anti-atherogenic factors. Further incubation of THP-1 cells with serum reduced C3b/iC3b deposition. Overall, our data indicate that apoE and FH interact with monocytic cells in a concerted action and this interaction reduces complement activation and inflammation in the atherosclerotic lesions. By this way FH may participate in mediating the beneficial effects of apoE in suppressing atherosclerotic lesion progression.

## Introduction

Complement (C) system is part of the humoral innate immune response. It quickly attacks microbes and foreign particles invading the human body. Activation of complement through any of the three pathways, the classical, alternative (AP), and lectin pathways, leads to cleavage of C3 and covalent surface deposition of the C3b fragment that is capable of forming an enzyme with factor B. Surface deposited C3b and its fragments, inactive C3b (iC3b) and C3dg, are important opsonins that can be recognized by complement receptors expressed by phagocytes. Further activation of the C cascade leads to formation of membrane attack complexes and release of proinflammatory chemotactic and anaphylatoxic protein fragments C3a and C5a that mediate their effects by binding to receptors on phagocytes.

AP is constantly active in plasma leading to low grade challenge to all plasma-exposed particles and surfaces by spontaneous hydrolysis or enzymatic activation of C3. Activation proceeds rapidly through amplification on surfaces that are missing efficient regulatory mechanisms. Factor H (FH) is the main AP regulator as it keeps this spontaneous activation in control (1). This is obvious since depletion of plasma/serum from FH or blockage of FH by autoantibodies leads to activation of the AP leading to overconsumption and loss of active complement within <30 min (2, 3). FH is an elongated molecule composed of 20 domains. The N-terminal domains 1–4 are responsible for FH regulatory activity while domains 19–20 on the C-terminal end are responsible for the self surface recognition (4). Domain FH19 binds to surface-deposited C3b, while FH20 binds sialic acids and glycosaminoglycans (GAGs) present on self surfaces (5). In this way, FH discriminates host self surfaces from non-self ones. Also domains 6–7 of FH are important for self surface recognition. These mediate interaction with sulphated GAGs, heparin, and C-reactive protein (6, 7). When bound to C3b, the cofactor activity of FH helps in inactivation of C3b to iC3b by factor I and simultaneous inhibition of factor B binding to C3b (8, 9). Moreover, FH prevents further AP amplification by accelerating the decay of formed AP convertases (10). AP activation does not need a trigger as it is based on continuous low-grade activity. However, imbalance between activation and regulation e.g., when numerous C3b deposits are formed on protein complexes can lead to enhanced AP activation in serum/plasma (11). Recently, it has become clear that AP dysregulation is a central event in development of several complement-related diseases to which mutations or polymorphisms in domains FH5-7 and FH19-20 predispose. While mutations in FH19-20 cause atypical hemolytic uremic syndrome (aHUS), the Y402H polymorphism in domain 7 is associated with age-related macular degeneration (AMD) (12, 13) and dense deposit disease (DDD) (14).

Atherosclerosis is a chronic multifactorial inflammatory disease caused by the subendothelial accumulation of lipids, immune cells and fibrous elements in arteries leading to thickening and hardening of the arterial wall. Low-grade inflammation is a key mediator of the disease. Both adaptive and innate immune responses are crucial for initiation and progression of atherosclerosis, and these mechanisms have been exploited to develop new diagnostic biomarkers and therapies for patients recently (15). The hallmark of early atherosclerotic lesion is the formation of fatty streaks composed of cholesterol-laden macrophages, which are formed when low density lipoproteins (LDL) are modified by oxidation or proteolytic modification and accumulated in the subendothelium of arteries leading to monocyte recruitment and differentiation into macrophages (16). The balance between proinflammatory M1 type macrophages and anti-inflammatory M2 type macrophages plays a crucial role in the pathogenesis of atherosclerotic plaques.

The antiatherosclerotic activity of apoE is based on its ability to regulate lipoprotein metabolism and to promote cholesterol efflux from cells (17, 18). Endogenous production of apoE by macrophages in blood vessel walls has been shown to be critical in the prevention and healing of atherosclerotic plaques. Importantly, apoE modulates macrophage polarization into the anti-inflammatory M2 phenotype (17) and promotes reverse cholesterol transport from peripheral cells to high density lipoprotein (HDL) for further transportation of cholesterol to the liver for excretion (19).

Genetic variations in the *APOE* gene coding for apolipoprotein E constitute important risk factors both for AMD and atherosclerosis. Interestingly, similar underlining mechanisms including disturbances in lipid metabolism, oxidative stress and the inflammatory process are closely associated in the pathogenesis of both diseases. It has also been shown that in human eyes with AMD, FH co-localizes with and binds to oxidized lipids in drusen, fatty deposits under the retina. It seems that the common FH variant 402Y has a higher affinity for oxidized lipids than the risk allele 402H suggesting a stronger FH-mediated complement inhibition of the effects of oxidized lipids on macrophages (20).

We have shown before that FH binds both lipid-free and high density lipoprotein (HDL) associated apoE via domains 5–7 and thereby regulates AP activation in plasma (21). The present study was set up to investigate whether FH and apoE interaction could play a role in the induction and progression of atherosclerosis by macrophages. We show here that FH increases apoE binding to monocytes and THP-1 macrophages possibly via simultaneous interaction between cell surface sialic acids and apoE and thereby regulates local complement activation. Moreover, FH interaction with THP-1 macrophages and cholesterol-labeled cells increases macrophage-mediated cholesterol efflux and modulates the expression of inflammatory genes suggesting a yet unexplored anti-inflammatory mechanism for FH.

## Materials and methods

### Proteins

Cloning and *Pichia pastoris* expression of the recombinant fragments FH5-7, FH19-20, and FH1-4 has been described earlier (22, 23). If necessary, fragments were further purified by passing through a HiLoad 16/60 Superdex 200 prep-grade gel filtration column (GE healthcare) in phosphate buffered saline (NaCl 300mM, KCl 5.4 mM, Na2HPO4 20 mM, KH2PO4 3.6 mM, pH 7.4), and concentrated using heparin affinity chromatography. Labeling of proteins was performed using N-hydroxysuccinimide-reactive Red dye (NT647, catalog no. L001) following the manufacturer's instructions (NanoTemper).

### Expression of apoE proteins

The preparation of vectors, expression of recombinant apoE2, apoE3, and apoE4 in the *Escherichia coli* BL21-Gold (DE3) bacterial system following induction by IPTG, and purification by immobilized metal affinity chromatography has been described elsewhere (24–26).

### Isolation of HDL and LDL and acetylation of human LDL

LDL (d = 1.019–1.050 g/mL) and HDL (d = 1.063–1.210 g/mL) were isolated from plasma of healthy volunteers obtained from the Finnish Red Cross Blood Service by sequential ultracentrifugation using KBr for density adjustment (27). LDL was acetylated by repeated additions of acetic anhydride (28). Briefly, LDL (10 mg as LDL protein) in 1.5 ml LDL buffer (150 mM NaCl, 1 mM EDTA, pH 7.4) was mixed 1:1 (vol/vol) with saturated sodium acetate and stirred in ice-water bath for 10 min. Next 30 μl acetic anhydride was added four times with 10 min stirring intervals. After the fourth addition of acetic anhydride, the incubation was continued for 60 min with continuous stirring. Finally, the mixture was dialysed for 24 h at 4°C against LDL buffer.

### Isolation of peripheral blood cells, cell cloning, and culturing

For peripheral blood cell isolation blood was drawn to tubes containing hirudin (Roche Diagnostics, Mannheim, Germany) from healthy human volunteers after informed written and signed consent (Ethical Committee decision 406/13/03/00/2015, Hospital district of Helsinki and Uusimaa). The blood samples were diluted 1:1 (v/v) with PBS and centrifuged through a gradient (Histopaque® 1.119 and 1.077; Sigma-Aldrich) at 320 x g for 20 min at 22°C. The PBMC layer was collected, washed once with RPMI 1640 (Gibco®) containing 0.05% (w/v) HSA (RPMI-HSA) and diluted with RPMI-HSA. U937 human monocytic cells were obtained from the ATCC (American Type Culture Collection), cultured in RPMI medium supplemented with penicillin/streptomycin and 10% (v/v) FCS. CR3 was stably expressed in U937 cells using a lentiviral expression system as described (29). We cloned the CD11b (NM_001145808.1) and CD18 (NM_000211.4) cDNA in the dual promoter lentiviral vectors, RP137 (BIC-PGK-Zeo) and RP-139 (BIC-PGK-Puro), respectively. These vectors were constructed by replacing the Zeo-T2a-mAmetrine cassette from the BIC-PGK-Zeo-T2a-mAmetrine (RP172) vector (30) with either a Zeocin or Puromycin resistance gene. First CD11b was stably expressed in U937 cells, subsequently these cells were used for stable expression of CD18. THP-1 monocytes were transformed to macrophages by incubating the cells for 48–72 h in the presence of 100 nM phorbol 12-myristate 13-acetate (PMA) and for generating cholesterol loaded cells mimicking foam cells the macrophages were further incubated in the presence of 100 μg/ml (as LDL protein) acetylated LDL for 24–48 h in serum free media.

### Detection of CR3 and sialic acid expression on cells and binding of FH on cell surface sialic acids

The expression of CR3 was analyzed by incubating the cells in 50 μl RPMI-HSA at 5 × 10^6^ cells/ml concentration for 45 min with FITC-conjugated anti-CD11b (sigma). The cells were washed once with RPMI-HSA and expression of CR3 was detected by flow cytometry. To detect the specificity of sialic acid expression on different cells and FH binding to cell surface sialic acids the cells were preincubated with 100U α2-3,6,8 Neuraminidase (New England biolabs) or PBS for 30 min at 37°C. Next, 45 nM NT647 labeled Maackia Amurensis Lectin II (MAL II^*^) or 200 nM NT647-FH was added to the wells and the plate was further incubated for 30 min at 37°C. The cells were washed once with 200 μl of icecold RPMI-HSA, centrifuged at 300 × g for 10 min, fixed with 100 μl of 1% (v/v) paraformaldehyde (Thermo) RPMI-HSA. Next, 2,000–10,000 cells were run by BD LSR Fortessa flow cytometer (Lazer 640 nm, filter 670/30) and analyzed using FlowJo V10 software, where the gating of the cells was performed by using forward scatter (FSC) and side scatter (SSC) to find viable, single cell events. Mean fluorescence intensities were calculated for the gated cells.

### ApoE, FH5-7 and factor H binding to cells

Binding of FH or FH5-7 to peripheral blood monocytic cells, U937 and THP-1 cells was studied by incubating 2 × 10^5^ cells with 200 nM of NT647-FH or 1,3 μM of NT647 FH5-7 for 45 min at 4°C in round bottom 96-well plates. For inhibition assays, cells were incubated for 30 min at 4°C with 1.5 μM of apoE, FH1-4 or 9 and 3 μM of FH5-7 prior adding the labeled protein. The cells were washed once with 200 μl of icecold RPMI-HSA, centrifuged at 300 × g for 10 min and fixed with 100 μl of 1% (v/v) paraformaldehyde RPMI-HSA. To study the effect of apoE-FH interaction and their binding to monocytic cells a dilution series of FH (Complement technologies), FH19-20 or apoE was incubated in dark for 5 min at 37°C with a constant 200 nM concentration of NT647-labeled apoE or NT647-labeled FH. Then 40 μl of hirudin blood isolated monocytes were added in each well and incubated in dark for 25 min at 37°C. The incubation was stopped by adding 300 μl of ice-cold PBS to the tubes and the cells were run by flow cytometry and analyzed using the gating strategy described earlier.

### FH5-7 binding to a panel of leukocyte receptors

Monocytes isolated from peripheral blood (PBMCs) cells (5 × 10^6^ cells/ml) in RPMI-HSA 0.05% (w/v) were incubated in the presence of 14 μg/ml of FH5-7, or PBS for 15 min on ice followed by incubation with a panel of receptor specific FITC-, PE- or APC-labeled antibodies using a 96-well U-plate (greiner, Bio one) for 45 min at 4°C. The cells were washed with 200 μl of RPMI/HSA 0.05% (w/v) and fixed with 150 μl of 1% (v/v) paraformaldehyde in RPMI-HSA 0.05% for 30 min at 4°C before counting the fluorescence of 10,000 cells using on FACS flow cytometer (Lazers 405, 488, and 640 nm; filters 450/50, 525/50, and 670/30) and analyzed using the gating strategy described earlier. The inhibition value of FH5-7 on receptor specific antibody binding was calculated by dividing the mean fluorescence of FH5-7 treated cells by PBS treated cells.

### C3b/iC3b deposition on FH incubated THP-1 cells

Untreated THP-1 monocytes, PMA activated THP-1 macrophages, and PMA activated THP-1 macrophages loaded with cholesterol by using acLDL (100/ml μg of LDL protein) were incubated in the presence or absence of FH (320 nM) for 24 h in 24-well tissue culture plates at 37°C in 5% CO_2_ in serum free THP-1 medium using 4 x 10^5^ cells/well/200 μl. A 10 μl sample from the supernatant was taken at 0, 5 and 24 h timepoints after the media was centrifuged for 10 min at 300 x g to remove any cellular debris. These culture media aliquots were stored at −20 °C before analysis for the apoE ELISA method. After 24 h the cells were detached using 200 μl Cellstripper (Corning) for 45 min at 37°C, 5% CO_2_, harvested by centrifugation as before and diluted with RPMI-HSA. Cells from this assay were used to analyze NT647-FH binding (described earlier), mRNA expression, apoE secretion (described later), ABCA1 protein expression and complement activation assays. To detect ABCA1 protein expression the cells were incubated with 0.3 μg/4 x 10^5^ cells/well/150 μl mouse anti-ABCA1 antibody (abcam) washed once by centrifugation and incubated with 1:200 dilution of Alexa Fluor 488 conjugated goat anti-mouse IgG (Invitrogen). To compare serum complement activity in the presence or absence of preincubated FH 5 x 10^4^ of THP-1 cells were incubated for 15 min in a 50 μl volume of 20% (v/v) serum. Cells were washed once by centrifugation and incubated with 2 μl of FITC conjugated anti-C3b (Cederlane) for 45 min at 4°C. The cells were washed, fixed, run by flow cytometry (Lazer 488, filter 525/50) and analyzed as described earlier.

### Effect of FH on apoE secretion and binding to THP-1 cells

The cells detached from the tissue culture 24-well plates were incubated in the presence of 2 μl of rabbit anti-human apoE (600 μg/ml, non affinity purified) in a 50 μl volume of RPMI-HSA 0.05% (w/v) for 45 min at 4°C. The cells were washed once by centrifugation and incubated in the presence of 1:100 diluted 488 goat anti-rabbit antibody (Life technologies) for 45 min at 4°C. After incubation the cells were washed, fixed and analyzed by flow cytometry as described earlier (Lazer 488, filter 525/50). Secretion of apoE by THP-1 cells was analyzed from 8 μl of culture media collected at different time points using apoE ELISA protocol as previously described (31).

### Cholesterol efflux assay

The cholesterol efflux assay was performed according to manufacturer's instructions (Abcam) using THP-1 cells activated for 24 h with PMA. The cells were labeled overnight with fluorescently-labeled cholesterol in the presence or absence of 650 nM FH. After overnight labeling the PMA activated and cholesterol labeled THP-1 macrophages were washed gently with 200 μL of RPMI media and incubated then in the presence of 50 μg of HDL (as HDL protein) as cholesterol acceptor. After 5 h incubation the media and supernatant of lysed cells were measured separately for fluorescence (Ex/Em = 482/515 nm). The ratio between fluorescence intensity of media and fluorescence intensity of cell lysate plus media were calculated as percentage of cholesterol efflux.

### RNA-sequencing

RNA sequencing method was designed based on the Drop-seq protocol described earlier (32). Briefly, the cells were mixed with lysis buffer (0.3% (v/v) triton, 20 mM DTT, 2 mM dNTPs) in wells of U-bottomed 96-well plate. Magnetic Dynabeads (M-270 Streptavidin, Thermo Fisher Scientific) coated with Indexing Oligonucleotides (Integrated DNA Technologies, Table 1) were added to each well. After 5 min of incubation at ambient temperature the magnetic beads were separated from the supernatant and washed twice with 6X SSC buffer. Subsequently, the beads were combined with RT mix, containing 1 x Maxima RT buffer, 1 mM dNTPs, 10 U/μl Maxima H- RTase (all ThermoFisher Scientific), 1 U/μl RNase inhibitor (Lucigen), and 2.5 μM Template Switch Oligo (Integrated DNA Technologies). Samples were incubated in a T100 thermal cycler (BioRad) for 30 min at 22°C and 90 min at 52°C. The beads were washed twice with 6X SSC buffer and once in PCR-grade water. The constructed cDNA was amplified by PCR in a volume of 15 μl using 5 μl of RT mix as template, 1x HiFi HotStart Readymix (Kapa Biosystems) and 0.8 μM SMART PCR primer. The samples were thermocycled in a T100 thermocycler (BioRad) as follows: 95°C 3 min; subsequently four cycles of: 98°C for 20 s, 65°C for 45 s, 72°C for 3 min; following 13 cycles of: 98°C for 20 s, 67°C for 20 s, 72°C for 3 min; and with the final extension step of 5 min at 72°C. The PCR products were pooled together and purified with 0.6X Agencourt AMPure XP Beads (Beckman Coulter) according to the manufacturer's instructions. They were eluted in 10 μl of molecular grade water. The 3′-end cDNA fragments for sequencing were prepared using the Nextera XT (Illumina) tagmentation reaction with 600 pg of the PCR product serving as an input. The reaction was performed according to manufacturer's instruction, with the exception of the P5 SMART primer that was used instead of S5xx Nextera primer. Subsequently, the samples were PCR amplified as follows: 95°C for 30 s; 11 cycles of 95°C for 10 s, 55°C for 30 s, 72°C for 30 s; with the final extension step of 5 min at 72°C. Samples were purified twice using 0.6X and 1.0X Agencourt AMPure Beads (Beckman Coulter) and eluted in 10 μl of molecular grade water. The concentration of the libraries was measured using a Qubit 2 fluorometer (Invitrogen) and the Qubit DNA HS Assay Kit (ThermoFisher Scientific). The quality of the sequencing libraries was assessed using the LabChip GXII Touch HT electropheresis system (PerkinElmer), with the DNA High Sensitivity Assay (PerkinElmer) and the DNA 5K/RNA/Charge Variant Assay LabChip (PerkinElmer). Samples were stored at −20°C. The libraries were sequenced on Illumina NextSeq500, with a custom primer (Table 1) producing read 1 of 20 bp and read 2 (paired end) of 55 bp (32). Sequencing was performed at the Functional Genomics Unit of the University of Helsinki, Finland.

**Table 1:** Primers used in this study.

**Name**	**Oligonucleotide sequence**
DSbI 001	TTTTTTTAAGCAGTGGTATCAACGCAGAGTACACGTACGTACGTNNNNNNNNTTTTTTTTTTTTTTTTTTTTTTTTTTTTTT
DSbI 002	TTTTTTTAAGCAGTGGTATCAACGCAGAGTACCGTACGTACGTANNNNNNNNTTTTTTTTTTTTTTTTTTTTTTTTTTTTTT
DSbI 003	TTTTTTTAAGCAGTGGTATCAACGCAGAGTACGTACGTACGTACNNNNNNNNTTTTTTTTTTTTTTTTTTTTTTTTTTTTTT
DSbI 004	TTTTTTTAAGCAGTGGTATCAACGCAGAGTACTACGTACGTACGNNNNNNNNTTTTTTTTTTTTTTTTTTTTTTTTTTTTTT
DSbI 005	TTTTTTTAAGCAGTGGTATCAACGCAGAGTACACGTCGTACGTANNNNNNNNTTTTTTTTTTTTTTTTTTTTTTTTTTTTTT
DSbI 006	TTTTTTTAAGCAGTGGTATCAACGCAGAGTACCGTAGTACGTACNNNNNNNNTTTTTTTTTTTTTTTTTTTTTTTTTTTTTT
DSbI 007	TTTTTTTAAGCAGTGGTATCAACGCAGAGTACGTACTACGTACGNNNNNNNNTTTTTTTTTTTTTTTTTTTTTTTTTTTTTT
DSbI 008	TTTTTTTAAGCAGTGGTATCAACGCAGAGTACTACGACGTACGTNNNNNNNNTTTTTTTTTTTTTTTTTTTTTTTTTTTTTT
DSbI 009	TTTTTTTAAGCAGTGGTATCAACGCAGAGTACACGTGTACGTACNNNNNNNNTTTTTTTTTTTTTTTTTTTTTTTTTTTTTT
DSbI 010	TTTTTTTAAGCAGTGGTATCAACGCAGAGTACCGTATACGTACGNNNNNNNNTTTTTTTTTTTTTTTTTTTTTTTTTTTTTT
DSbI 011	TTTTTTTAAGCAGTGGTATCAACGCAGAGTACGTACACGTACGTNNNNNNNNTTTTTTTTTTTTTTTTTTTTTTTTTTTTTT
DSbI 012	TTTTTTTAAGCAGTGGTATCAACGCAGAGTACTACGCGTACGTANNNNNNNNTTTTTTTTTTTTTTTTTTTTTTTTTTTTTT
TSO	AAGCAGTGGTATCAACGCAGAGTGAATrGrGrG
SMART PCR primer	AAGCAGTGGTATCAACGCAGAGT
P5 SMART primer	AATGATACGGCGACCACCGAGATCTACACGCCTGTCCGCGGAAGCAGTGGTATCAACGCAGAGT^*^A^*^C
Sequencing read 1	GCCTGTCCGCGGAAGCAGTGGTATCAACGCAGAGTAC

* = phosphorothioate bond added

### Read alignment and generation of digital expression data

Raw sequence data was filtered to remove reads shorter than 20 bp. Subsequently, the original pipeline suggested in Macosko et al. (32) for processing of drop-seq data was used. Briefly, reads were additionally filtered to remove polyA tails of length 6 or greater, then aligned to the human (GRCh38) genome using STAR aligner (33) with default settings. Uniquely mapped reads were grouped according to the 1–12 barcode, and gene transcripts were counted by their Unique Molecular Identifiers (UMIs) to reduce the bias emerging from the PCR amplification. Digital expression matrices (DGE) reported the number of transcripts per gene in a given sample (according to the distinct UMI sequences counted).

### Statistical methods

Statistical analyses between multiple samples were performed using one-way ANOVA supplemented with non-parametric Tamhane's multiple-comparison test. Statistical differences between two independent samples were calculated using non-parametric Mann-Whitney *U*-test (SPSS for Windows, Analytical Software).

## Results

### Factor H binds to monocytic cell CR3 via CD11b

**Figure 1:**
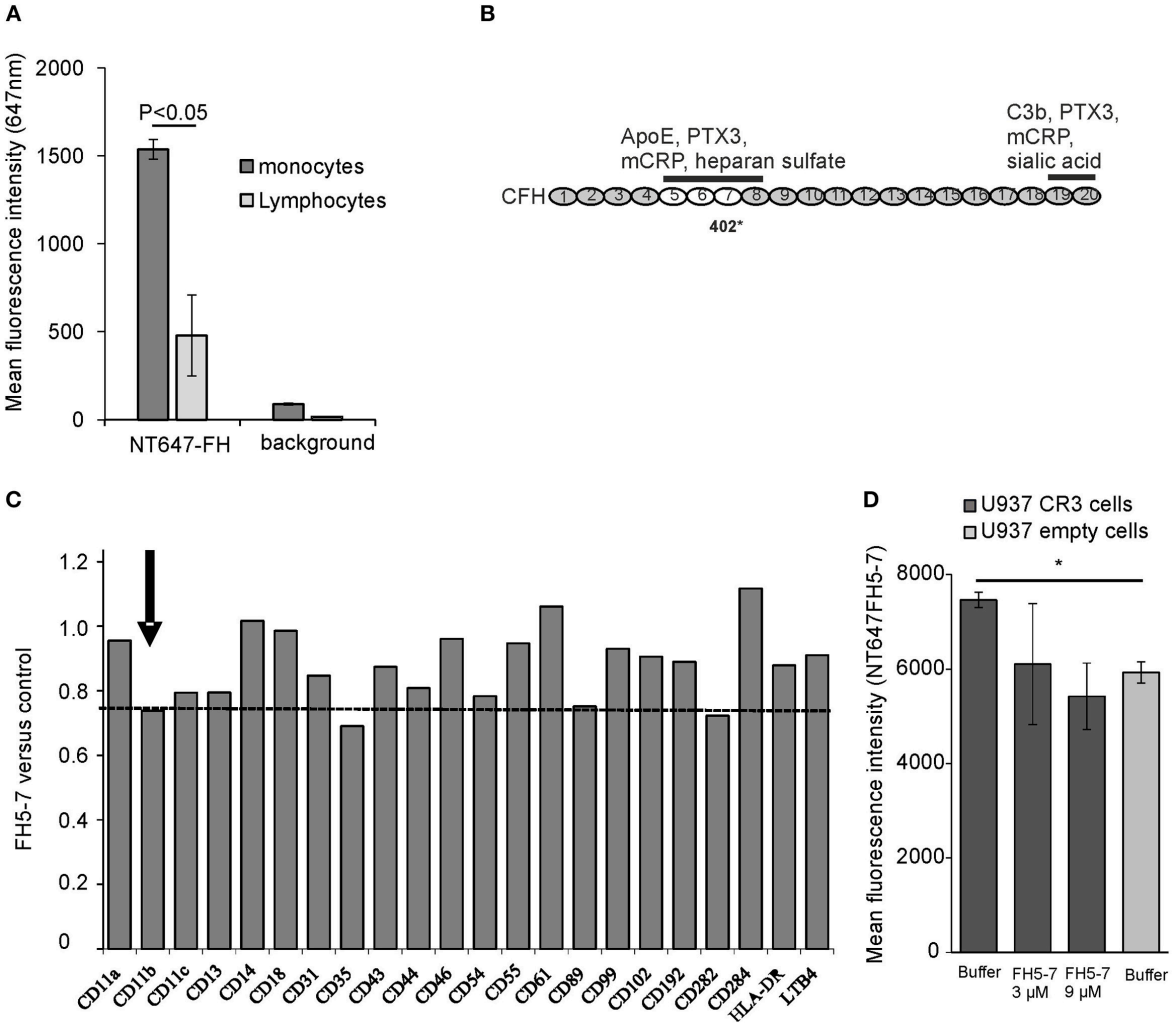
Factor H binding to peripheral blood leukocytes. **(A)** Peripheral blood mononuclear cells were incubated with NT647-labeled FH and the mean fluorescence intensities were analyzed by flow cytometry (*n* = 3). **(B)** Schematic structure of the full length FH and the location of domains 5–7 that interact with apoE. The binding sites of different ligands, apoE, pentraxin3 (PTX3), monomeric C-reactive protein (mCRP) and heparin sulfate, and the common polymorphic Y402H site on domain 7 are shown. **(C)** Screening of FH5-7 binding by a panel of antibodies directed against various receptors and surface molecules on monocytes. Peripheral blood monocytic cells were preincubated for 30 min with or without 12 μg/ml of FH5-7 prior adding 2 × 10^5^ cells cells/well in a 96-well plate with FITC-, APC- and/or PE-labeled antibody. Mean fluorescence intensities were calculated from the gated cells using flow cytometry. Inhibition of antibody binding was calculated by dividing the mean fluorescence of FH5-7 treated cells by PBS treated cells (FH5-7 vs. control). The CD11b part of CR3 dimer is marked. The dashed line shows the level of anti-CD11b binding in the presence of FH5-7 (< 0.8) compared to binding of the antibody without FH5-7 (1.0). **(D)** CR3 expressing and empty U937 mononuclear cells were incubated with NT647 labeled FH5-7 in the presence or absence of unlabeled FH5-7 (x-axis). Statistical significance between multiple samples was calculated using one-way ANOVA supplemented with non-parametric Tamhane's *post-hoc* multiple comparison test. Error bars indicate SD. ^*^ = *p* < 0.05.

FH interacts with human cell surfaces mainly via C3b and cell surface glycosaminoglycans, like heparan sulfate, and sialic acids. Previous studies have shown that especially on endothelial cells and platelets the presence of sialic acids is crucial for efficient FH-mediated complement regulation (34). In addition to surface sialic acids, FH has been shown to interact with CR3 (CD11b/CD18) on human neutrophils in the absence of C3b deposits (35). We studied binding of FH to different ligands on monocytes and monocytic cell lines to find out how these interactions could be altered by apoE that is known to be secreted by macrophages and to interact with FH domains 5–7 (21, 36). By using NT647-labeled FH we could detect clear binding of FH to monocytes even in the absence of C3b, while binding of FH to lymphocytes was closer to the background values (Figure 1A). To find out the receptor that interacts with the apoE binding domains FH5-7 (location of the apoE binding domains is shown in Figure 1B) on monocytes we performed a screening assay where the cells were preincubated with the recombinant FH5-7 fragment prior to adding the anti-receptor antibody. Incubation of the cells with FH5-7 resulted in a clear reduction in anti-CD11b binding that is the alpha-chain of the integrin-type CR3 receptor heterodimer (CD11b/18; Figure 1C). In addition, similar inhibition was also detected with antibodies against CD35 and CD89. Binding of FH5-7 on CR3 was further analyzed using CR3 overexpressing U937 monocytic cells (Figure 1D). Binding of NT647-labeled FH5-7 on these cells was reduced in the presence of increasing concentrations of non-labeled FH5-7 (3 and 9 μM) and on empty U937 cells that do not express CR3.

### Binding of apoE to monocytes is increased by factor H

**Figure 2:**
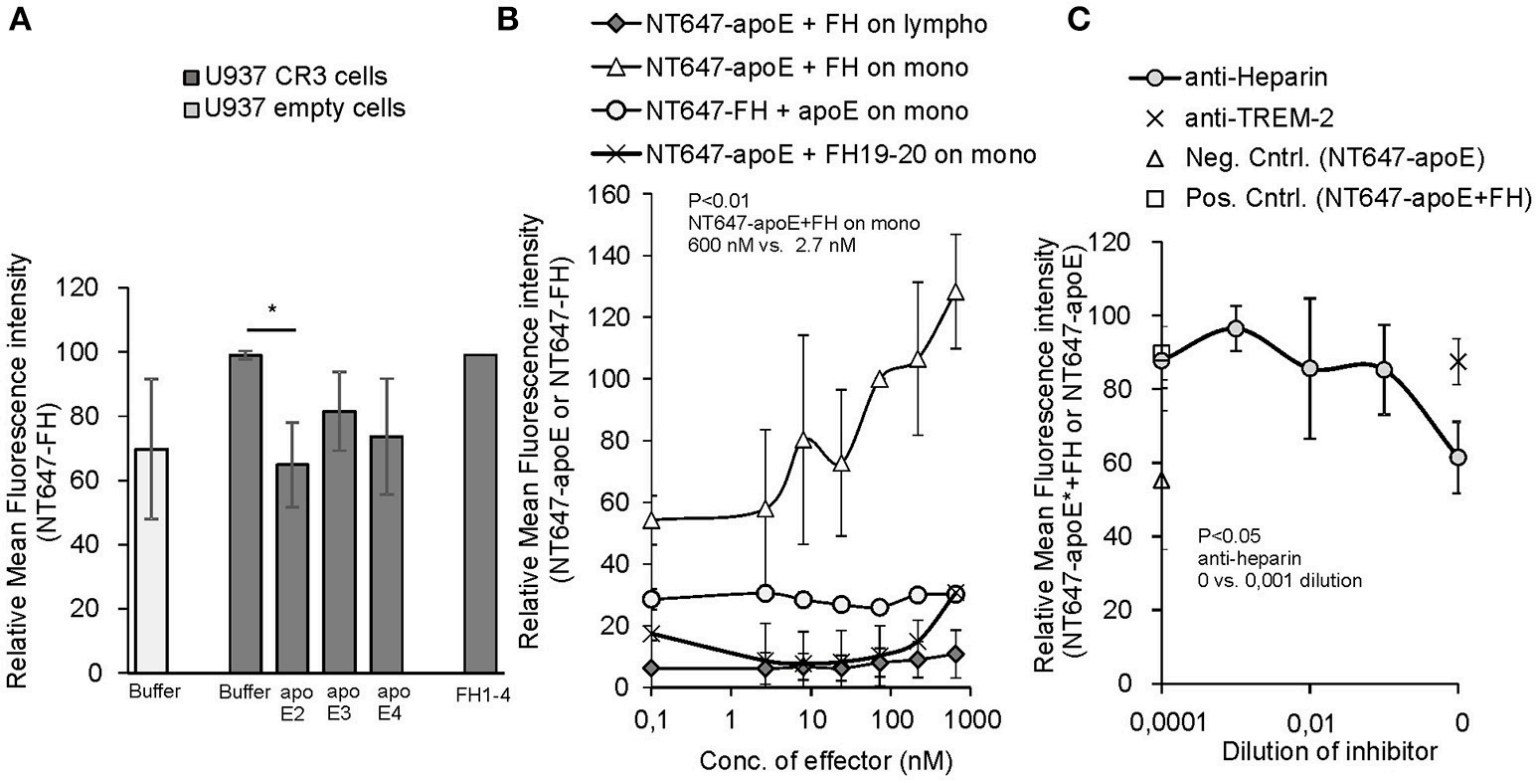
Inhibition of Factor H/apoE binding to U937 and peripheral blood cells. **(A)** Binding of NT647-FH on U937 cells expressing CR3 in the absence (Buffer) or presence of 1.5 μM of apoE2, 3 and 4 (*n* = 3). Control cells without CR3 (empty cells) shows binding of FH without the receptor. Binding of NT647-FH in the presence of equal molarity of FH1-4 is shown. **(B)** Binding of NT647 labeled FH or apoE2 on peripheral blood cells with different concentrations of unlabeled inhibitor (FH or apoE). Levels are calculated as relative to apoE binding at 73 nM FH concentration (*n* = 4). **(C)** Inhibition of apoE+FH binding to monocytes in the presence of increasing concentrations of anti-heparin antibody. Anti-TREM-2 antibody was used as a negative control. Control (Neg. Cntrl. NT647-apoE) shows binding of apoE on the cells without FH incubation and Control (Pos. Cntrl. NT647-apoE+FH) binding of apoE in the presence of FH and apoE only (*n* = 3). Statistical significance was calculated using one-way ANOVA supplemented with non-parametric Tamhane's *post-hoc* multiple comparison test. Error bars indicate SD. Percentages of mean fluorescence intensities are shown as relative to the maximum intensity in each individual experiment. ^*^= *p* < 0.05.

To study further the interaction between the apoE binding ligand FH and CR3 on human monocytes, we next performed an assay to see whether apoE could alter FH-CR3 interaction on U937 monocytic cells overexpressing the receptor. We found that on CR3 overexpressing U937 cells binding of NT647-FH was clearly inhibited by apoE2 to the level of binding of FH to cells devoid of CR3. A slight inhibition was also detected by apoE3 and apoE4 but the levels of inhibition were not statistically significant (Figure 2A). As the apoE2 isotype showed significant inhibition we used the apoE2 isoform in the further assays. Surprisingly, when the same inhibition assay was performed using peripheral blood monocytes and lymphocytes we did not detect any inhibition of NT647-FH binding in the presence of increasing concentrations of unlabeled apoE (Figure 2B). On the contrary, a clear increase in binding of NT647-apoE was detected when the cells were incubated with increasing concentrations of unlabeled FH, while FH19-20 did not have the same effect. This indicates that FH increases rather than attenuates apoE binding to monocytes. To further study the cell surface receptors leading to increased binding of apoE to these cells, and because both apoE and FH are known to interact with cell surface heparan sulfate, we incubated monocytes with NT647-apoE and FH and increasing concentrations of anti-heparin antibody. Here, binding of NT647-apoE in the presence of anti-heparin antibody was reduced to the level of NT647-apoE only (Figure 2C) suggesting that increased binding of apoE to these cells is dependent on FH and cell surface heparan sulfate.

**Figure 3:**
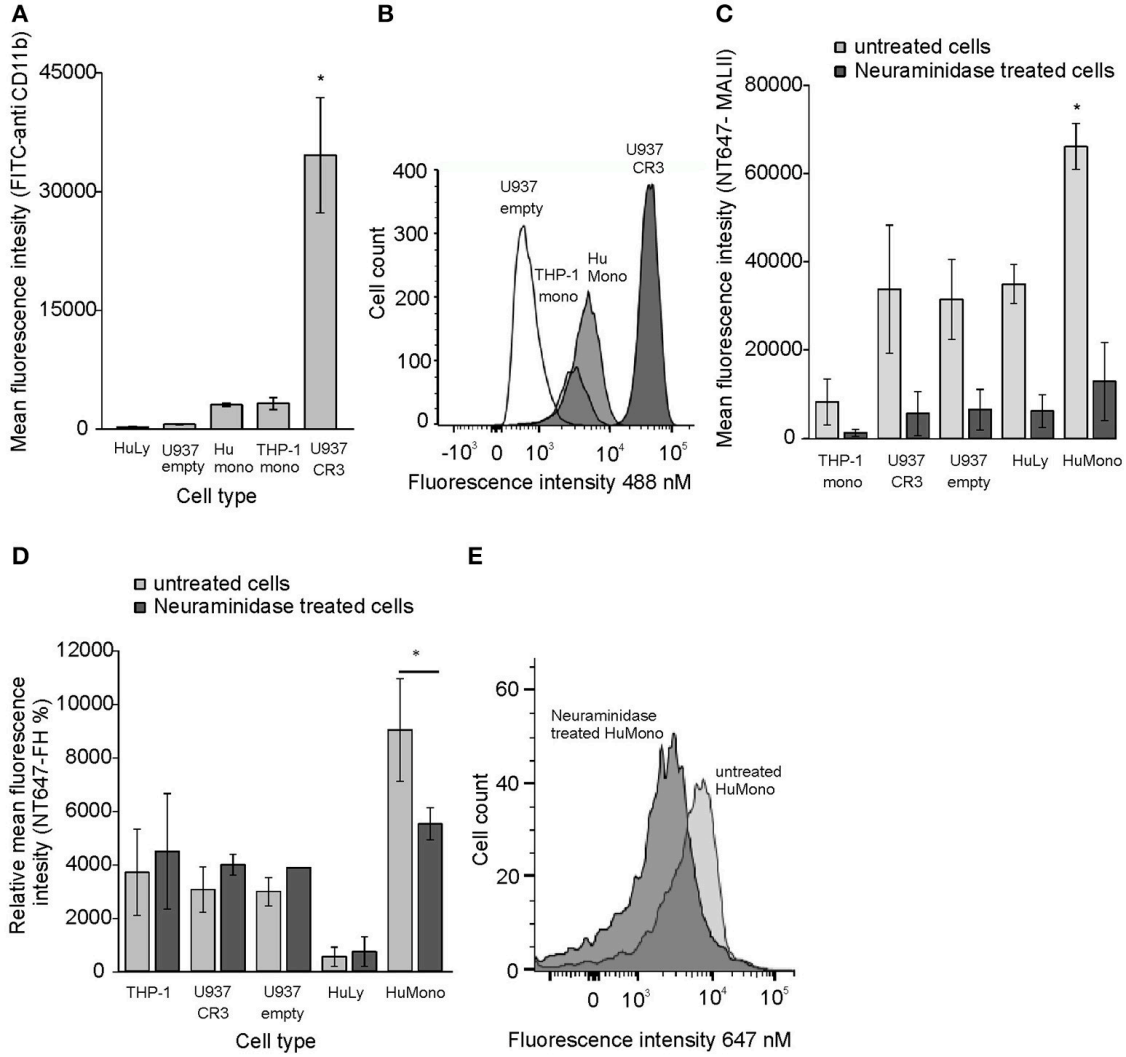
CR3 and sialic acid expression and FH binding to neuraminidase treated cells. **(A)** The expression of CR3 in different cell types was tested using fluorescent labeled anti-CD11b antibody and flow cytometric analysis (*n* = 4). **(B)** Histogram showing distribution of CR3 receptors in different cell types. **(C)** Binding of NT647 labeled Maackia amurensis lectin I (MAL II) to cell surface sialic acids in the presence or absence of 100 U neuraminidase (*n* = 3). **(D)** Binding of NT647 labeled FH on cell surface sialic acids in the presence or absence of 100 U neuraminidase (*n* = 3). **(E)** Binding of NT647 labeled FH on monocyte surface sialic acids in the presence or absence of 100 U neuraminidase presented in a histogram. Percentages of mean fluorescence intensities is shown as relative to the intensity of neuraminidase treated U937 cells in each individual experiment. Histogram showing binding of NT647-FH on different cell types. Statistical significance between multiple samples was calculated using one-way ANOVA supplemented with Tamhane's *post-hoc* multiple comparison test. Statistical significance between two samples was calculated using Mann-Whiney *U*-test. Error bars indicate SD. ^*^ = *p* < 0.05.

Because we could detect inhibition of FH binding by apoE only to U937 cells overexpressing CR3 but not to monocytes we hypothesized that on monocytes, where CR3 expression is lower, FH could simultaneously bind to apoE and cell surface sialic acids. This is because FH sialic acid binding domains are located within domains FH19-20 and not on apoE interacting domains FH5-7. This could also explain why apoE binding was increased by FH to monocytes. As expected, the human peripheral blood monocytes showed high sialic acid expression and low CR3 expression while the U937 cells showed very low expression of sialic acids and very high CR3 expression (Figures 3A–C). The high expression levels of CR3 and low levels of sialic acids on U937 cells may explain why inhibition of FH binding to CR3 in the presence of apoE could only be detected on these cells. To study the effect of sialic acids on binding of FH different cell types were preincubated with neuraminidase that removes cell surface sialic acids. All tested cells bound FH at different levels, but only monocytes showed significant reduction in FH binding after neuraminidase treatment. No decrease in FH binding was detected on other tested cell types with lower sialic acid expression (Figures 3D,E). These data suggest that FH interacts with several different ligands on cells but binding of FH to sialic acids via domains FH19-20 enables simultaneous binding of apoE via domains FH5-7 and cell surface heparan sulfate.

### Binding of FH to THP-1 cells reduces complement activation

**Figure 4:**
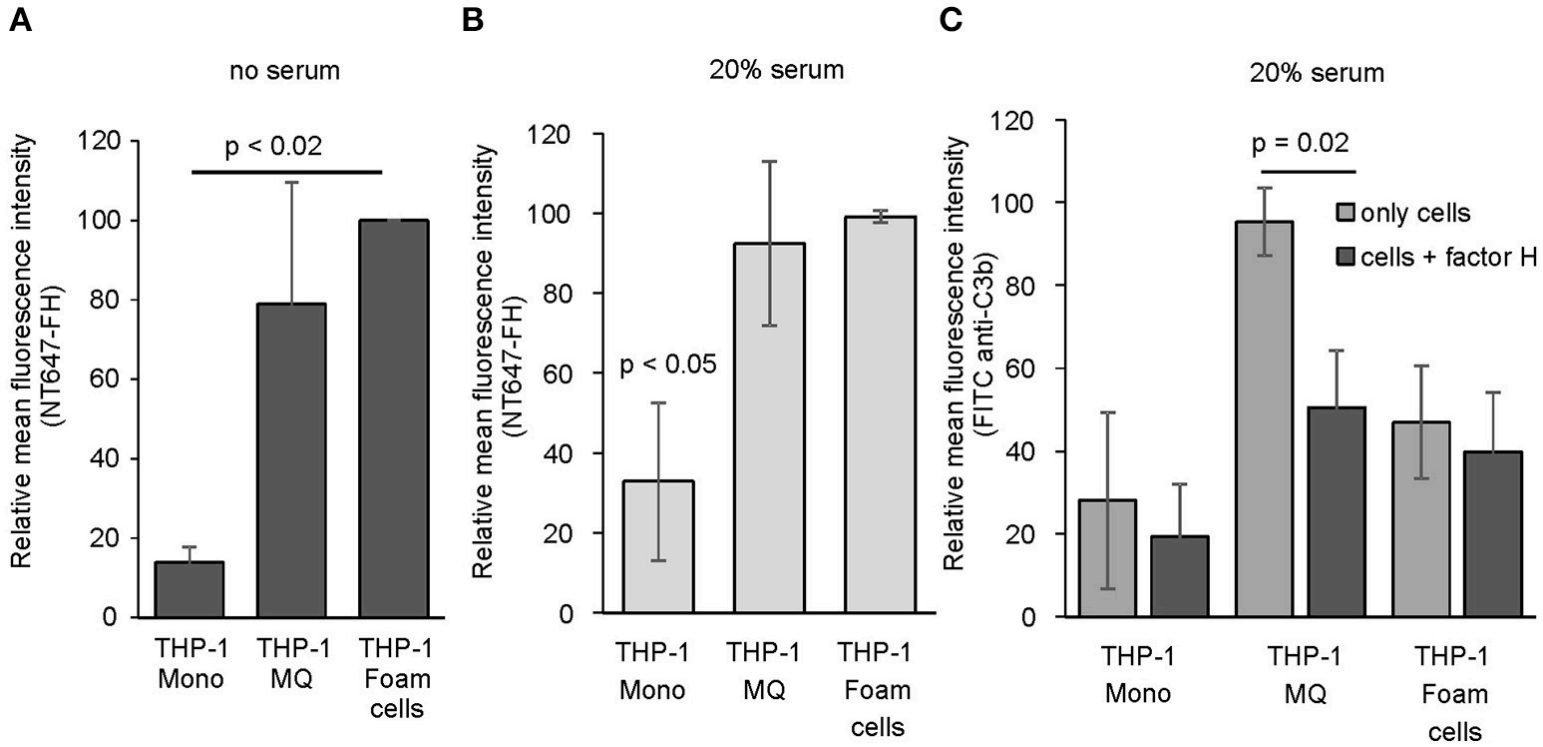
Binding of FH and C3b deposition on THP-1 cells. Binding of NT647-labeled FH to THP-1 monocytes, THP-1 macrophages and cholesterol-loaded THP-1 macrophages (foam cells) in the **(A)** absence (*n* = 3) or **(B)** presence (*n* = 3) of 20% serum. **(C)** Deposition of serum C3b on THP-1 monocytes, THP-1 macrophages and THP-1 foam cells in the presence or absence of FH (*n* = 3). Statistical significance was calculated using using one-way ANOVA supplemented with Tamhane's *post-hoc* multiple comparison test. Error bars indicate SD. Percentages of mean fluorescence intensities are shown as relative to the maximum intensity in each individual experiment.

Since we found that FH interacts directly with human peripheral blood cells and because activation of complement is known to play a role in the induction and progression of atherosclerosis (37) we next studied whether FH binding to macrophages and cholesterol-loaded macrophages could have effect on local complement activation. To study this, we used THP-1 monocytes stimulated with PMA and acetyl LDL as model cells of early stage atherosclerosis. To avoid measuring FH binding to damaged cells the viability was determined using Trypan blue staining. These cell populations had cell viability of approximately 70%. When THP-1 monocytes, THP-1 macrophages and acLDL (i.e., cholesterol) loaded THP-1 macrophages were studied for NT647-FH binding in the absence of serum, a significant increase in FH binding was detected on activated cholesterol loaded THP-1 macrophages compared to THP-1 monocytes (Figure 4A). When the cells were incubated with NT647-FH in the presence of 20% serum a similar trend in FH binding could be detected indicating that the increase in FH binding due to THP-1 activation is independent from surface C3b deposition. Both THP-1 macrophages and cholesterol loaded macrophages showed a significant increase in FH binding compared to THP-1 monocytes (Figures 4A,B). To study whether increased FH binding to these cells has functional significance in reducing local complement activation, the serum incubated cells were also analyzed for C3b deposition. Surprisingly, preincubation of THP-1 cells in the presence of FH showed a clear reduction in cell surface C3b deposition only in the case of THP-1 macrophages (Figure 4C). Incubation of THP-1 monocytes or cholesterol-loaded THP-1 cells in 20% serum did not lead to an increase in cell surface C3b deposition. Therefore, the presence of additional FH had an effect on the cell targeted complement activity only on THP-1 macrophages.

### FH increases apoE binding to THP-1 cells and macrophage-mediated cholesterol efflux

**Figure 5:**
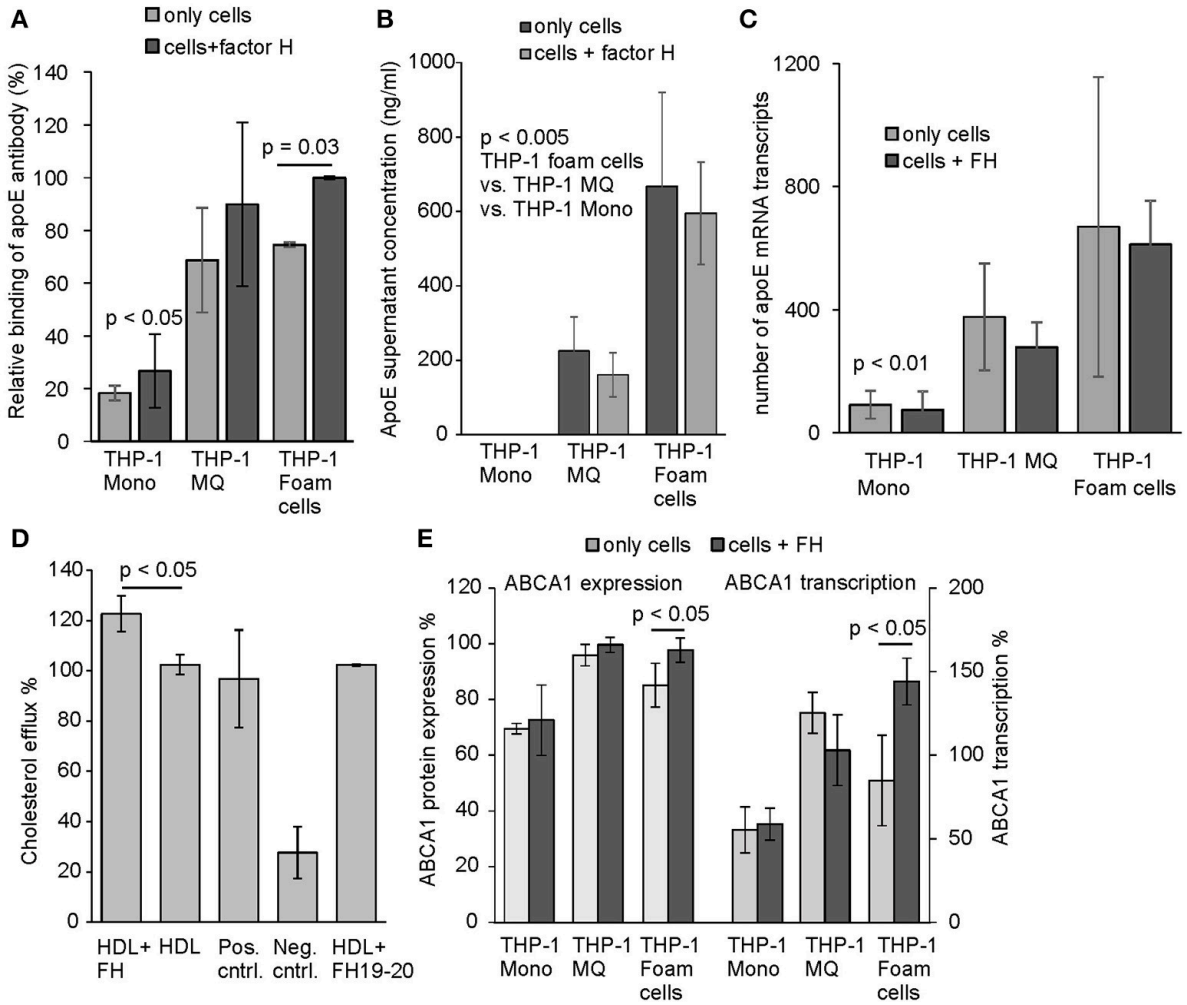
Binding, secretion, and expression of apoE and cholesterol efflux by THP-1 cells. **(A)** Surface expression of and/or binding of apoE from culture supernatants to THP-1 monocytes, THP-1 macrophages and cholesterol-loaded THP-1 cells detected by anti-apoE antibody. Cells were incubated with and without FH for 24 h. Next, the cells were washed and detached from the tissue culture plates. Presence of apoE on cell surfaces were detected using anti-human apoE and Alexa 488 labeled goat anti-rabbit antibody in flow cytometry. ApoE binding is shown as relative to the maximum intensity in each individual experiment (*n* = 3). **(B)** Secretion of apoE to cell culture media by THP-1 monocytes, THP-1 macrophages and cholesterol-loaded THP-1 cells detected by ELISA (*n* = 4). **(C)** Number of apoE mRNA transcripts analyzed by sequencing the cell isolated mRNA (*n* = 4) **(D)** Cholesterol efflux from non-loaded THP-1 macrophages labeled with fluorescent cholesterol in the presence or absence of FH (*n* = 3). Cholesterol efflux in the presence of equal molarity of FH19-20 is shown. The positive (Pos. cntrl. from Abcam) and non HDL treated controls (Neg. cntrl.) were included in the assay. **(E)** Protein expression and transcription levels of ABCA1 in THP-1 monocytes, THP-1 macrophages and cholesterol-loaded THP-1 cells detected by anti-ABCA1 antibody and mRNA sequencing. Levels are calculated as relative to the protein expression and transcription in THP-1 macrophages (*n* = 4). Statistical significance calculated using Mann-Whiney *U*-test or one-way ANOVA supplemented with Tamhane's multiple comparison test. Error bars indicate SD.

We used anti-apoE antibody to detect surface bound apoE on THP-1 cells incubated with or without FH under cell culture conditions. Similarly to the human peripheral monocytes apoE binding was detected to activated THP-1 cells from which cholesterol loaded THP-1 macrophages demonstrated significant increase in apoE binding in the presence of FH, while binding of apoE on THP-1 monocytes was low (Figure 5A). This correlated well with the previous results, where cholesterol loading of the cells resulted in most significant FH binding. This indicates that FH binding to THP-1 cell surfaces increases apoE binding as well. Because endogenous production of apoE by macrophages in blood vessel wall has been suggested to promote healing of atherosclerotic plaques and efficient transport of cholesterol out from the cell (17), we analyzed the amount of secreted apoE in the culture media. After a 24 h incubation apoE secretion increased significantly from activated THP-1 cells among which cholesterol-loaded THP-1 cells showed highest apoE concentrations in the culture media (Figure 5B). These results also correlated well with the transcriptome data obtained from the apoE mRNA sequencing performed from the isolated cells after 24 h incubation (Figure 5C). No difference between cells incubated either in the presence or absence of FH could be observed in apoE expression. ApoE is a good cholesterol acceptor among many other apolipoproteins having the potential to bind phospholipids ensuring cholesterol removal (38). Therefore, we next studied whether elevated apoE binding via FH to THP-1 cells could affect cholesterol efflux. Here, a significant increase in cholesterol efflux could be detected from cholesterol-labeled THP-1 macrophages that were incubated in the presence of FH when compared to cells in the absence of FH (Figure 5D).

### FH increases transcription of anti-inflammatory genes in macrophages

While cholesterol loaded THP-1 macrophages showed highest apoE secretion FH did not have any effect on this. We, however, hypothesized that FH could display anti-inflammatory effects on macrophages as it interacts with both macrophages and cholesterol loaded cells, inhibits complement activity on macrophages, increases cholesterol efflux from cholesterol labeled THP-1 macrophages and increases apoE interaction with these cells as well. After 24 h incubation the THP-1 cell mRNAs were isolated and subjected to sequencing-based transcriptome analysis covering over 27,000 RNA sequences of different genes. Transcriptome analysis resulted in a selection of transcripts that are associated with inflammation, atherosclerosis and the complement system [Table 2, (18, 39–46)]. When these transcriptomes were compared between the FH and PBS incubated cells some clear effects/differences could be seen between THP-1 macrophages and cholesterol loaded THP-1 macrophages. As expected and based on the above FH and apoE binding assays THP-1 monocytes remained unresponsive to FH treatment. FH treatment resulted in increase of ABCA1 transcription in cholesterol loaded macrophages (foam cells) and in a significant increase in transcription of a regulator of ABCA1 expression, PPAR-α (40). Similarly, FH increased ABCA1 expression as well and correlated with the significantly increased transcription levels of the protein (Figure 5E). In THP-1 macrophages the expression of proinflammatory factors CX3CR1, CCL5, and SAAL1 was significantly decreased by FH. Moreover, a reduction in transcription of complement receptor C5aR2 by FH in macrophages was detected although this was not statistically significant (*p* = 0.053).

**Table 2:** Changes in the transcriptome in response to FH in human THP-1 cells.

**Immunological function**	Up(+)/ down (–) regulation^**^	**Function**	Gene^*^	**THP-1 Foam cells**			**THP-1 MQ**			**THP-1 Mono**		
				**FH**	**PBS**	***p*-value**	**FH**	**PBS**	***p*-value**	**FH**	**PBS**	***p-*value**
Anti-inflammatory	+	Nrf2 has a role in resistance to oxidant stress. Effect on atherogenesis may vary depending on the activator (39, 40)	NFE2L2	176.8	76.9	**0.013**	131.9	171.3	0.386	142.1	171.7	0.556
Antiatherogenic	+	Transporter that controls apoAI-mediated cholesterol efflux from macrophages (41)	ABCA1	832.8	555.1	**0.075**	598.9	742.6	0.270	340.4	324.7	0.835
Antiatherogenic	+	PPAR-α and PPAR-γ activators induce the expression ABCA1 (42)	PPARA	18.3	3.5	**0.032**	11.6	26.1	0.211	16.4	18.1	0.925
Clearance	+	The protein levels of RIPK1/3 are positively correlated with the extent of necroptosis (43)	RIPK1	39.8	3.5	**0.004**	20.3	43.7	0.053	25.4	9.2	0.071
Proinflammatory	±	Knockdown of SLC17A9 significantly suppres both M1-type polarization and IL-6 production (44)	SLC17A9	113.8	58.6	**0.039**	58.5	122.7	**0.033**	187.2	131.7	0.242
Proinflammatory	–	CX3CL1 is a chemokine involved in the adhesion and migration of leukocytes.	CX3CR1	0.0	3.5	0.356	0.0	13.0	**0.001**	6.8	12.4	0.633
Proinflammatory	–	CCL5 promotes macrophage recruitment and survival in human adipose tissue (45)	CCL5	32.9	32.7	0.990	5.3	27.8	**0.005**	31.1	10.1	0.145
Proinflammatory	–	promotes proliferation of fibroblasts upon inflammation (46)	SAAL1	8.8	0.0	**0.027**	1.9	16.4	**0.006**	3.8	2.0	0.618
Proinflammatory	+	receptor for IL-3 IL-5 GMCSF	CSF2RB	29.4	0.0	**0.000**	22.2	14.8	0.614	17.4	22.1	0.789
Proinflammatory	~	Binds C5a, involved in coronary artery disease and in pathogenesis of sepsis (18)	C5AR2	400.1	267.2	0.114	438.1	612.9	**0.053**	115.0	132.5	0.684
	No effect	Binds C5a	C5AR1	193.3	215.6	0.564	158.9	142.5	0.779	47.1	24.2	0.273
	No effect	apolipoprotein E	APOE	669.2	611.6	0.828	375.9	278.4	0.415	90.8	74.6	0.684
	No effect	factor H	CFH	0.0	0.0		0.0	5.1	0.214	0.0	0.0	

* Panel of genes known to play a role in inflammation or atherosclerosis with the description of their function in inflammation. The numbers show the amount of mRNA transcripts (= mRNA expression level).

** *Up- and downregulation marked as + and – and filled with dark or light gray colors, respectively. Statistical significance (p-values < 0.05 marked in bold numbers) calculated between cells incubated with or without FH using Student's t-test. Gray numbers = no statistical significance between FH and PBS treated cells*.

## Discussion

In the present study we showed that complement FH increases apoE binding to macrophages and leads to an increased cholesterol efflux and reduced inflammation. The FH/apoE interaction is hypothesized to limit the progression of atherosclerosis as complement regulation by FH is critical in the prevention self-cell damage and exacerbated inflammation.

We previously found that FH interacts with apoE and reduces complement activation in plasma HDL particles (21). The current study shows that this interaction apparently limits inflammation in the atherosclerotic lesions by affecting macrophage activation and cholesterol efflux. FH was found to interact with cell surface sialic acids and CR3 on monocytic cells in the absence of surface deposited C3b. In the presence of apoE the interaction between FH and CR3 was inhibited, while apoE had no effect on cells abundantly expressing sialic acids. In addition to CR3, our screening assay suggested that domains 5–7 of FH could also interact with CD35 (C3b/C4b receptor, CR1) and CD89. From these, CR3 and CR1 are receptors directly involved in C-mediated clearance and suppression of inflammation. We have previously shown that FH blocks binding of CR1 to C3b (47) as both molecules compete for the same binding site on C3b. Like FH, CR1 is also a C regulator and therefore should be separately analyzed for its role in C regulation during the atherosclerotic lesion development. CR1 is mostly present on cell surfaces but may also occur in soluble form. Its main function is the transport of immune complexes and other unwanted materials for clearance by macrophages in the spleen and liver.

Factor H binding to cell surface C3b increases in the presence of self cell glycosaminoglycans and sialic acids. The binding of FH to CR3 has still been regarded as controversial, probably because of the possibility of C3b contamination in the sample. The novelty we show here in the regard of FH and CD11b interaction is that in the absence of C3b FH binding to CR3 expressing cells can be inhibited by apoE2 indicating a common binding site between apoE and CR3 on FH domains 5–7. Inhibition of FH binding to these cells by apoE3 and apoE4 was not that obvious indicating that the single amino acid differences (Cys and Arg in positions 112 and 158) between the apoE isotypes could alter binding to FH. Analogous differences between these isoforms have been observed earlier due to their structural variation. For instance, apoE3 and apoE4 protein isoforms bind well to LDL-receptors, whereas apoE2 displays defective binding (48). CR3 is known to interact with several ligands. A major ligand that promotes phagocytosis is iC3b on opsonised microbes and other particles. According to the mRNA expression data obtained from FH stimulated cells it is unlikely that FH could trigger CR3-mediated signaling, although a weak but not significant increase in CLEC7A (dectin-1) expression in cholesterol-loaded THP-1 macrophages could be detected in the presence of FH (Table 2). This C-type lectin is upregulated in anti-inflammatory M2 macrophages (49). However, earlier studies have shown that binding of FH on CD11b suppresses acute subretinal inflammation in mice indicating that FH-CR3 interaction could reduce inflammation in humans as well (50).

In contrast to the lacking inhibitory effect of apoE on FH-monocyte interaction, the presence of FH increased apoE binding to these cells significantly. This observation could be due to the high abundance of sialic acids and low expression levels of CR3 on these cells. Sialic acids favor FH binding to the cell surface via domains 19–20 enabling simultaneous interaction with apoE via domains 5–7. The effect of sialic acids on FH binding was demonstrated by neuraminidase treatment of different cell types, where only monocytes showed a clear reduction in FH binding at different cell surface sialic acid densities. Moreover, binding of FH on THP-1 macrophages clearly reduced C3b deposition on these cells suggesting that FH binding to these cells prior to C3b deposition has an effect on local complement activation. As demonstrated by our mRNA expression data, FH expression on all THP-1 cell types was almost or completely absent, while apoE expression was clearly increased both by THP-1 macrophages and cholesterol-loaded cells.

We observed that FH did not induce secretion or expression of apoE by the studied cells but slightly reduced apoE concentrations in the culture media. However, FH significantly increased binding of apoE especially to cholesterol-loaded THP-1 cells that also showed the highest FH binding. According to trypan blue staining the increase in FH binding was not due to cell damage as the cell viabilities were the same in the absence and presence of FH. It is possible that this kind of rebinding or apoE capturing on the macrophage surfaces could promote apoE recycling that has been suggested to reduce intracellular cholesterol accumulation and thereby prevent formation of foam cells. Previous studies have shown that apoE can be spared from degradation in lysosomes and recycled to the cell surface in order to maximize cholesterol removal from the cell (41). The intracellular ATP binding cassette transporter ABCA1 is an important regulator of cellular cholesterol homeostasis. It is involved in apoE secretion and in lipidating apoE-containing particles secreted by macrophages. ABCA1 is suggested to promote apoE recycling as well. Our results on the effect of FH on cholesterol efflux suggest that the increased binding of apoE caused by FH could promote apoE recycling and thereby maximize cholesterol efflux from macrophages. Importantly, the mRNA expression data showed increase in ABCA1 expression on FH-treated cholesterol-loaded THP-1 macrophages and a significant increase in the expression of PPAR-α transcription factor that is known to induce ABCA1 expression. This clearly suggests an anti-atherogenic function for FH-apoE interaction (42).

We found that treatment of THP-1 cells for 24 h clearly altered transcription of several genes associated with inflammation, atherosclerosis, and the complement system (Table 2). These changes occurred mainly within PMA-differentiated THP-1 macrophages and cholesterol loaded THP-1 macrophages while THP-1 monocytes as well as non-loaded THP-1 macrophages were less responsive to the treatment. These data did not suggest any clear M1/M2 polarization but general trend was that FH reduced significantly transcription of proinflammatory genes in macrophages (such as CXCR3, CCL5, SAAL1) but increased transcription of antiatherogenic genes that are involved in enhanced lipid metabolism (such as ABCA1, PPAR-α) mainly in cholesterol loaded cells. FH did not only result in increase in transcription of ABCA1 in cholesterol loaded macrophages but also in ABCA1 protein expression and PPAR-α transcription. This indicates that FH may affect in macrophage cholesterol efflux via this transcription factor known to activate ABCA1-mediated cholesterol efflux in human macrophages (42). Therefore, the difference between the transcription levels cannot be explained by the instability of mRNA transcripts that does not always correlate with the corresponding levels of protein expression (51).

**Figure 6:**
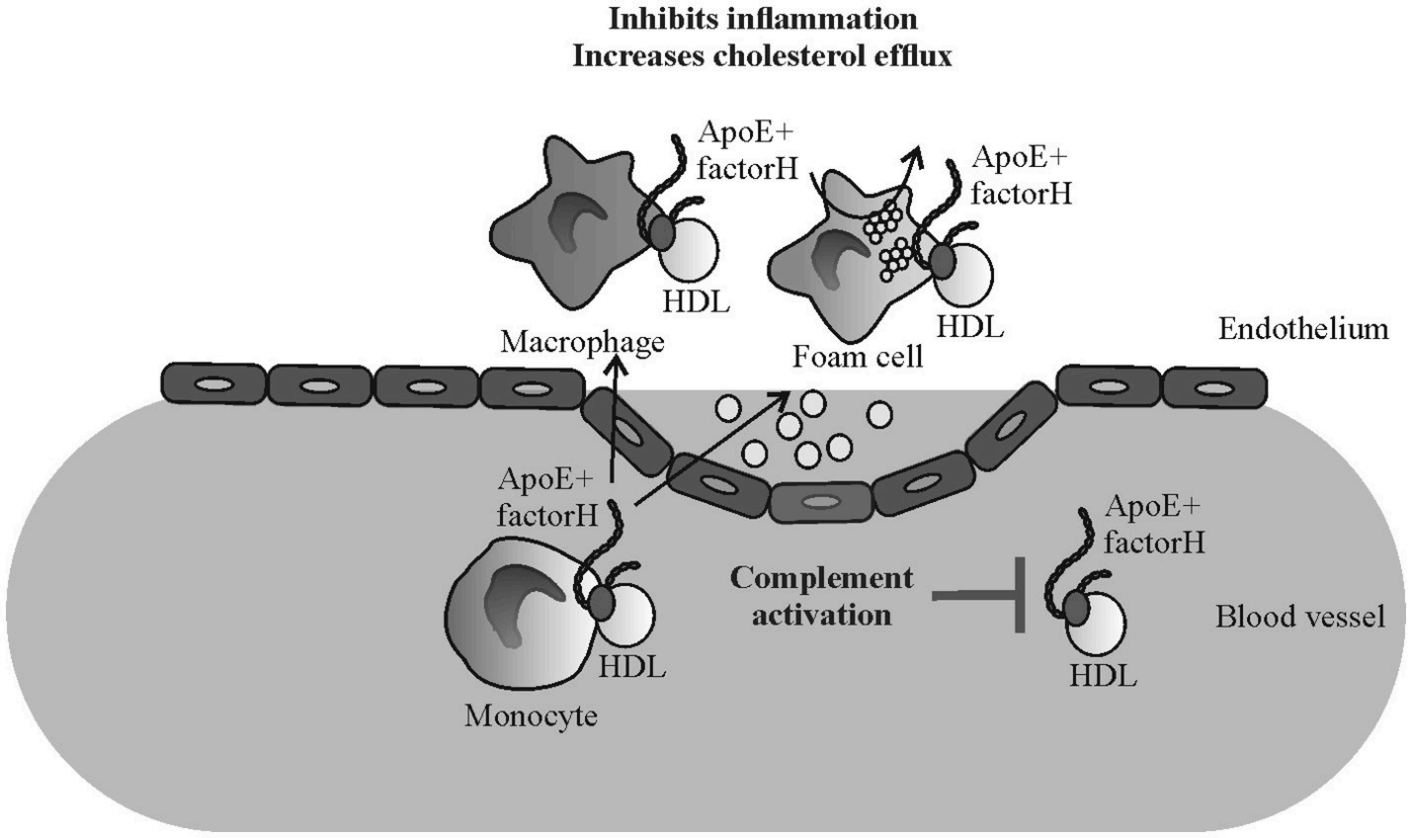
Schematic illustrating the putative mechanism of the effect of factor H-apoE interaction in reducing inflammation in atherosclerotic lesions. Binding of factor H on apoE containing HDL particles reduces plasma complement activation (21) while elevated binding of apoE on monocytes/macrophages/foam cells via FH might reduce local inflammation and cholesterol efflux.

Certain mutations and polymorphisms of FH are associated with AP dysregulation mediated diseases such as AMD, DDD, and aHUS. AMD and DDD are characterized by formation of deposits in the eye (drusen) or kidney (glomerular basement membrane) that are rich in complement activation products and, most importantly, also contain high amounts of apoE (52, 53). Therefore, in addition to atherosclerosis the pathogenesis of diseases such as AMD and DDD could be related to the FH and apoE-macrophage interactions. While FH interaction with apoE launches a concerted action leading to several anti-inflammatory responses on macrophages (Figure 6) genetic or acquired disturbances in this homeostatic mechanism could promote the progression of atherosclerotic and other analogous lesions.

## Author contributions

EN helped in data interpretation and manuscript evaluation, wrote the paper, and performed analysis. PH, KL, AR, SS, and JM performed analysis. JvS, KVK, CDH, and PS contributed data or analysis tools. KÖ, MJ, AC, SM, and SJ helped in data interpretation, helped to evaluate and edit the manuscript, contributed data or analysis tools. KH supervised development of work, helped in data interpretation and manuscript evaluation, designed the analysis, wrote the paper, and performed analysis.

### Conflict of interest statement

The authors declare that the research was conducted in the absence of any commercial or financial relationships that could be construed as a potential conflict of interest.
